# Differential Th17 response induced by the two clades of the pandemic ST258 *Klebsiella pneumoniae* clonal lineages producing KPC-type carbapenemase

**DOI:** 10.1371/journal.pone.0178847

**Published:** 2017-06-06

**Authors:** Ann Maria Clemente, Giuseppe Castronovo, Alberto Antonelli, Marco Maria D’Andrea, Michele Tanturli, Eloisa Perissi, Sara Paccosi, Astrid Parenti, Federico Cozzolino, Gian Maria Rossolini, Maria Gabriella Torcia

**Affiliations:** 1 Department of Experimental and Clinical Medicine, University of Firenze, Firenze, Italy; 2 Department of Medical Biotechnologies, University of Siena, Siena, Italy; 3 Department of Experimental and Clinical Biomedical Sciences, University of Firenze, Firenze, Italy; 4 Department of Health Science, University of Firenze, Firenze, Italy; 5 Clinical Microbiology and Virology Unit, Careggi University Hospital, Firenze, Italy; Louisiana State University, UNITED STATES

## Abstract

The spread of KPC-type carbapenemases is mainly attributed to the global dissemination of *Klebsiella pneumoniae* (KP) strains belonging to the clonal group (CG) 258, including sequence type (ST) 258 and other related STs. Two distinct clades of CG258-KP have evolved, which differ mainly for the composition of their capsular polysaccharides, and recent studies indicate that clade 1 evolved from an ancestor of clade 2 by recombination of a genomic fragment carrying the capsular polysaccharide (*cps*) locus. In this paper, we investigated the ability of two ST258-KP strains, KKBO-1 and KK207-1, selected as representatives of ST258-KP clade 2 and clade 1, respectively, to activate an adaptive immune response using *ex vivo*-stimulation of PBMC from normal donors as an experimental model. Our data showed that KKBO-1 (clade 2) induces a Th17 response more efficiently than KK207-1 (clade 1): the percentage of CD4^+^IL17^+^ cells and the production of IL-17A were significantly higher in cultures with KKBO-1 compared to cultures with KK207-1. While no differences in the rate of bacterial internalization or in the bacteria-induced expression of CD86 and HLA-DR by monocytes and myeloid dendritic cells were revealed, we found that the two strains significantly differ in inducing the production of cytokines involved in the adaptive immune response, as IL-1β, IL-23 and TNF-α, by antigen-presenting cells, with KKBO-1 being a more efficient inducer than KK207-1. The immune responses elicited by KK207-1 were comparable to those elicited by CIP 52.145, a highly virulent *K*. *pneumoniae* reference strain known to escape immune-inflammatory responses. Altogether, present results suggest that CG258-KP of the two clades are capable of inducing a different response of adaptive immunity in the human host.

## Introduction

*Klebsiella pneumoniae* producing KPC-type carbapenemases (KPC-KP) has emerged as an important cause of healthcare-associated infections correlated with high morbidity and mortality [1–3]. The clonal expansion of strains belonging to clonal group (CG) 258 (e. g. sequence type (ST) 258, ST512), and producing either the KPC-2 or the KPC-3 carbapenemases [4–10], has largely contributed to the pandemic diffusion of KPC-KP.

Recombination events in the genomic region coding for capsular polysaccharide (*cps* genes) led to the differentiation of two clades within members of CG258 [10,11]. The *cps* locus named *cps*-2 (or *cps*_KKBO-4_), likely derived from an ST442-like ancestor, identifies strains of clade 2, while the *cps* locus named *cps*-1 (or *cps*_KK207-2_), likely derived from an ST42 ancestor, identifies strains of clade 1 [10]. Capsular characterization of clinical isolates of KPC-KP, collected within a multicentre cross-sectional study promoted by the Italian Antibiotic-Resistance-Istituto Superiore di Sanità (AR-ISS) surveillance network, showed a predominance of clade 2 strains (88.8% of all CG258 isolates), and similar results are reported from the analysis of collections of CG258-KP from other geographic areas [12,13]. The reasons for the predominance of clade 2 strains remain to be clarified and, in this context, it is relevant to define the interactions of bacterial cells from the two clades with host immune system. What is known is that, compared with strains of clade 1, strains of clade 2 are endowed with a lower virulence potential in the *Galleria mellonella* model [14] and with a higher ability to activate the inflammatory pathways in cells of innate immunity [15].

The effective immune response during *K*. *pneumoniae* infection is mainly mediated by the production of interleukin (IL)-17 [16,17], a cytokine involved in neutrophil recruitment, and in the production of lipocalin-2 and of antimicrobial peptides by mucosal epithelial cells. The production of Interferon-γ (IFN-γ) also plays a role in the clearance of *K*. *pneumoniae* infections and, in particular, of those mediated by hypervirulent strains [18]. Although several innate cells (NKT and γδ T cells) are able to produce IL-17 and IFN-γ [19,20], a significant increase in the concentrations of these cytokines at the infection site is mainly induced by the activation of naïve or memory CD4^+^ T lymphocytes of T helper (Th)17 or of Th1 effector type. Antigen presenting cells usually drive the differentiation of Th17 or Th1 effectors through the production of IL-23 and IL-12 respectively [21]. IL-1β, TNF-α and IFN-γ further amplify the effectors differentiation [22,23].

In order to investigate the dominant type of adaptive immune response induced by the two clades of CG258-KP, we cultured representative strains of the two clades with circulating mononuclear cells (PBMC) from healthy donors and evaluated the differentiation/expansion of Th17 and Th1 CD4^+^ lymphocytes. The differentiation of T regulatory cells (Treg) was also studied.

## Materials and methods

### Antibodies and reagents

Anti-CD25-PE, anti-CD4-FITC, and anti-CD4-APC antibodies were obtained from BD Biosciences—Pharmingen (San Jose, CA, USA); anti-IL-17A-FITC, anti-IFN-γ-PerCP, and anti-Foxp3-APC (FJK-16s) staining kits were from eBiosciences (San Diego, CA, USA); anti-CD80-FITC, anti-CD86-APC and anti-HLA-DR-PE antibodies were obtained from BD Biosciences—Pharmingen. Phorbol 12 myristate 13-acetate (PMA), Ionomycin calcium salt and Brefeldin A (BFA) were from Sigma-Aldrich (Milano, Italy). Purified lipopolysaccharide (LPS) was purchased from Invivogen (San Giuliano Milanese, Milano, Italy). RPMI 1640, antibiotics (penicillin and streptomycin), L-glutamine, heat-inactivated fetal bovine serum were purchased from Celbio (Pero, Italy) and used for cultures with dendritic cells.

### *K*. *pneumoniae* strains: Properties and culture methods

Two KPC-KP strains of ST258, KKBO-1, and KK207-1, isolated in 2010 from two different Italian hospitals and epidemiologically unrelated to each other, were used [24,25]. These strains were selected as representative of clade 2 and clade 1 respectively based on the structure of their *cps* loci [24]. Whole genome sequences of KKBO-1 and KK207-1 (accession numbers: AVFC00000000 and LJOO00000000, respectively) demonstrated that these strains were closely related to other previously characterized representative strains of ST258 *K*. *pneumoniae* of the two clades including NJST258_2 and Kp1787, isolated in USA hospitals during outbreaks sustained by ST258-KP [10].

Besides the reported differences in the *cps* gene clusters [11,24], comparative genomic analysis of virulence genes content of KKBO-1 and KK207-1 revealed the presence, in both genomes, of a *mrkABCDFHIJ* operon that was not functional in KK207-1, due to an insertional inactivation of the *mrkH* gene.

The virulent strain of *K*. *pneumoniae* CIP 52.145 [26–28] was supplied by Pasteur Institute (Paris, France). CIP 52.145 is a derivative of B5055 (the reference strain of K2 serotype) belonging to ST66 and is genetically unrelated to ST258 strains according to the results of genome comparison. This strain, which expresses a number of virulence factors such as the RmpA plasmid-mediated regulator of mucoid phenotype and several siderophores [28,29], was used as a reference strain for its ability to escape molecular pathways relevant in the activation of the immune response [26,30]. All strains were grown overnight in Mueller-Hinton Broth (MHB), diluted 1:100 in MHB and reincubated at 37°C until an OD_600_ of 0.6 was reached.

Bacterial cells were inactivated by UV-light (20 minutes at 1 J/cm^2^) or by heating (95°C for 15 minutes) and used as antigen source at the indicated concentrations. Bacterial loss of viability was confirmed by CFU counting after incubation for 24 hours at 35±2°C on Mueller-Hinton agar plates.

### Isolation and culture of cells

The use of buffy coats from donated blood, not usable for therapeutic purposes, was approved by the Ethics Committee of the Azienda Ospedaliera Universitaria Careggi in agreement with the D.M. of Italian Ministry of Health (15A09709) G.U., n. 300 12.28. 2015). Buffy coats were collected at the Transfusional Center of the Azienda Ospedaliera Universitaria Careggi (Firenze, Italy) and peripheral blood mononuclear cells (PBMC) were isolated by gradient centrifugation using Ficoll-Paque (GE Healthcare Italia, Milan, Italy), according to the manufacturer’s recommendations.

CD14^+^ cells were isolated using anti-CD14 conjugated microbeads (Miltenyi Biotec, Bergisch Gladbach, Germany). To obtain monocyte-derived dendritic cells (MDC), CD14^+^ cells were cultured in the presence of 20 ng/ml of human recombinant (hr) IL-4 and 50 ng/ml of hr Granulocyte Macrophage Colony Stimulating Factor (GMCSF) (R&D Systems, Minneapolis, MN, USA) for seven days at 37°C in a humidified chamber with 5% CO_2_ [29]. MDC were recovered, plated at 10^6^ cells/ml and stimulated with bacterial cells.

### Endocytosis of bacterial cells

To determine the rate of bacterial endocytosis, 10^6^ monocytes or MDC were cultured with live bacterial cells (Multiplicity Of Infection (MOI) = 1) for 45 minutes. At the end of incubation, cells were centrifuged, washed with PBS and lysed with 0.1% triton X-100; bacterial cells, recovered from culture supernatant and cell lysates, were plated on Mueller-Hinton agar plates for CFU counts.

### Cell cultures and flow cytometry studies

Monocytes and MDC were cultured in RPMI medium supplemented with 10% fetal calf serum, 1% L-glutamine, 100 units of penicillin and 0.10 mg/mL of streptomycin (complete medium, CM) at 37°C in a humidified chamber with 5% CO_2_ with UV-inactivated or heat-inactivated bacterial cells at 1:10 ratio for the indicated times. Cells (5x10^5^) were stained with a mixture of anti-CD80, anti-CD86, and anti-HLA-DR labeled antibodies (0.5 μg/mL, Mix3) (Table 1), washed with cold PBS and analyzed by an ACCURI instrument (BD Biosciences, San Jose, CA, USA). Data were analyzed by CflowPlus software (BD Biosciences, San Jose, CA, USA). Ten thousand events for each sample were acquired.

**Table 1:** Conjugated antibodies used in this study.

	FITC	PE	PerCP	APC
**Mix1**	a-IL17A	a-CD25	a-IFN-γ	a-CD4
**Mix2**	a-CD4	a-CD25		a-FOXP3
**Mix3**	a-CD80	a-HLA-DR		a-CD86

To evaluate Th-differentiation induced by bacterial strains, PBMC (10^6^/well) were stimulated with UV-inactivated or heat-inactivated bacterial cells at 1:10 ratio in CM for 7 days.

Cells were recovered at the indicated times (0, 3, 5 and 7 days) and viability was assessed by Trypan blue exclusion. Cells were then stimulated with PMA (20 ng/ml)/Ionomicin (1μM) and Brefeldin (10 μg/ml) for 5 hours. Afterward, PBMC were stained with a mixture of labeled antibodies to anti-CD4-APC and anti-CD25-PE antibodies followed by intracellular staining with anti-IL-17A-FITC and anti-IFN-γ-PerCP antibodies (Mix1) (Table 1). To evaluate Treg differentiation, PBMC were stained with anti-CD4-FITC and anti-CD25-PE antibodies followed by intracellular staining with anti-Foxp3-APC (Mix2) (Table 1) [31].

Cells were analyzed by an ACCURI instrument and data were processed through CflowPlus software. Ten thousand events for each sample were acquired. The area of positivity was determined by using an isotype-matched control mAb.

### Cytokine measurement

IFN-γ, IL-1β, IL-6, IL-17, TNF-α, IL-10, IL-12 and IL-23 concentrations in culture supernatants of PBMC were determined by using a Milliplex kit (Millipore) and the BIOPLEX apparatus (Bio-Rad, Hercules, CA, USA), according to the manufacturer's recommendations as described in Clemente *et al*. [31].

### RT-PCR

Cellular RNA was extracted using TRIzol® Reagent (Invitrogen, Carlsbad, CA, USA) according to the manufacturer's recommendation. Reverse transcription of mRNA was performed using High-Capacity cDNA Reverse Transcription Kit (Applied Biosystems, Foster City, CA, USA). The amplification process was performed in triplicate with RT2 Real-Time™ SYBR Green / ROX PCR Master Mix (Applied Biosystems, Foster City, CA, USA) according to the manufacturer's recommendations on an ABI PRISM 7900 (Thermo Fisher Scientific, USA). Data were normalized to the mean value of house-keeping gene *GAPDH* mRNA and the relative amount of mRNA was calculated by using the 2^-ΔCT^ method

The nucleotide sequences of PCR Forward (Fw) and Reverse (Rv) primers are:

IL23p19:      Fw 5’-GCA GAT TCC AAG CCT CAG TC-3',

            Rv 5’-TTC AAC ATA TGC AGG TCC CA-3';

IL12p35:      Fw 5'-ACC ACT CCC AAA ACC TGC-3',

            Rv 5'-CCA GGC AAC TCC CAT TAG-3';

GAPDH:      Fw 5'-CACCATCTTCCAGGAGCGAG-3',

            Rv 5'-AAATGAGCCCCAGCCTTCTC-3';

### Statistical analysis

Statistical analysis was performed by Student’s *t*-test and One-way ANOVA with Tukey test for means comparison.

## Results

### Pathogen-induced production of cytokines in PBMC from healthy donors

As the first step of our study, we investigated the ability of *K*. *pneumoniae* strains to induce the production of inflammatory cytokines involved in the proliferation and differentiation of T-lymphocytes.

PBMC from healthy donors (n = 6) were cultured with UV-inactivated bacterial cells of the two ST258 KP strains (KKBO-1 or KK207-1) or of the CIP 52.145 strain, or with LPS as an internal standard, for 7 days. The concentration of IL-17A, IFN-γ, TNF-α, IL-1β, IL-6 and IL-10 was measured in the culture supernatants after 5 days of culture.

**Fig 1:**
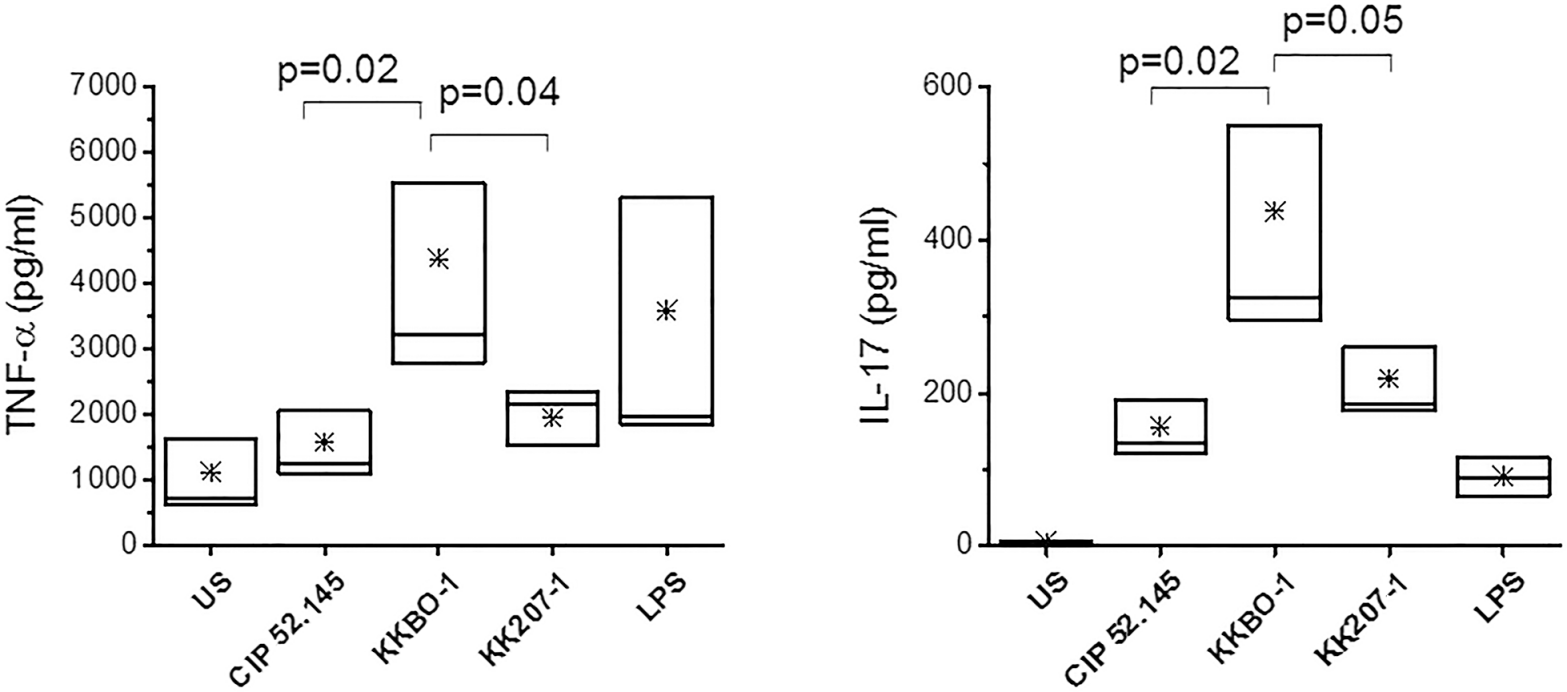
Cytokine production by PBMC stimulated with UV-inactivated bacterial cells. PBMC were isolated from 6 healthy donors and cultured at 10^6^/ml for 5 days with UV-inactivated bacterial cells from the ST258 KP-KPC strains or from CIP 52.145. LPS was used as an internal standard at 400 ng/ml. Cytokine concentration was measured in culture supernatants collected after 5 days of culture by Immunoplex array. The boxes extend from SE, with a horizontal line at the median. An asterisk indicates the mean value. Statistical analysis was performed by Student’s *t*-test and One-Way ANOVA and *p* ≤ 0.05 was considered significant.

Fig 1 showed that KKBO-1 strain significantly induces the production of IL-17A and TNF-α more than KK207-1 and CIP 52.145. It also induces the production of IFN-γ, IL-1β, IL-6 and IL-10 more than KK207-1 and CIP 52.145 but the differences among the strains did not reach statistical significancy (S1 Table). Comparable results were obtained by using heat-inactivated bacterial cells as a stimulus for cytokine production (S1 Fig).

### Pathogen-induced Th-differentiation

To assess the predominant type and the kinetic of T-cell differentiation induced by *K*. *pneumoniae* strains, we cultured PBMC from 6 different donors with UV-inactivated bacterial cells at 1:10 cell ratio.

**Fig 2:**
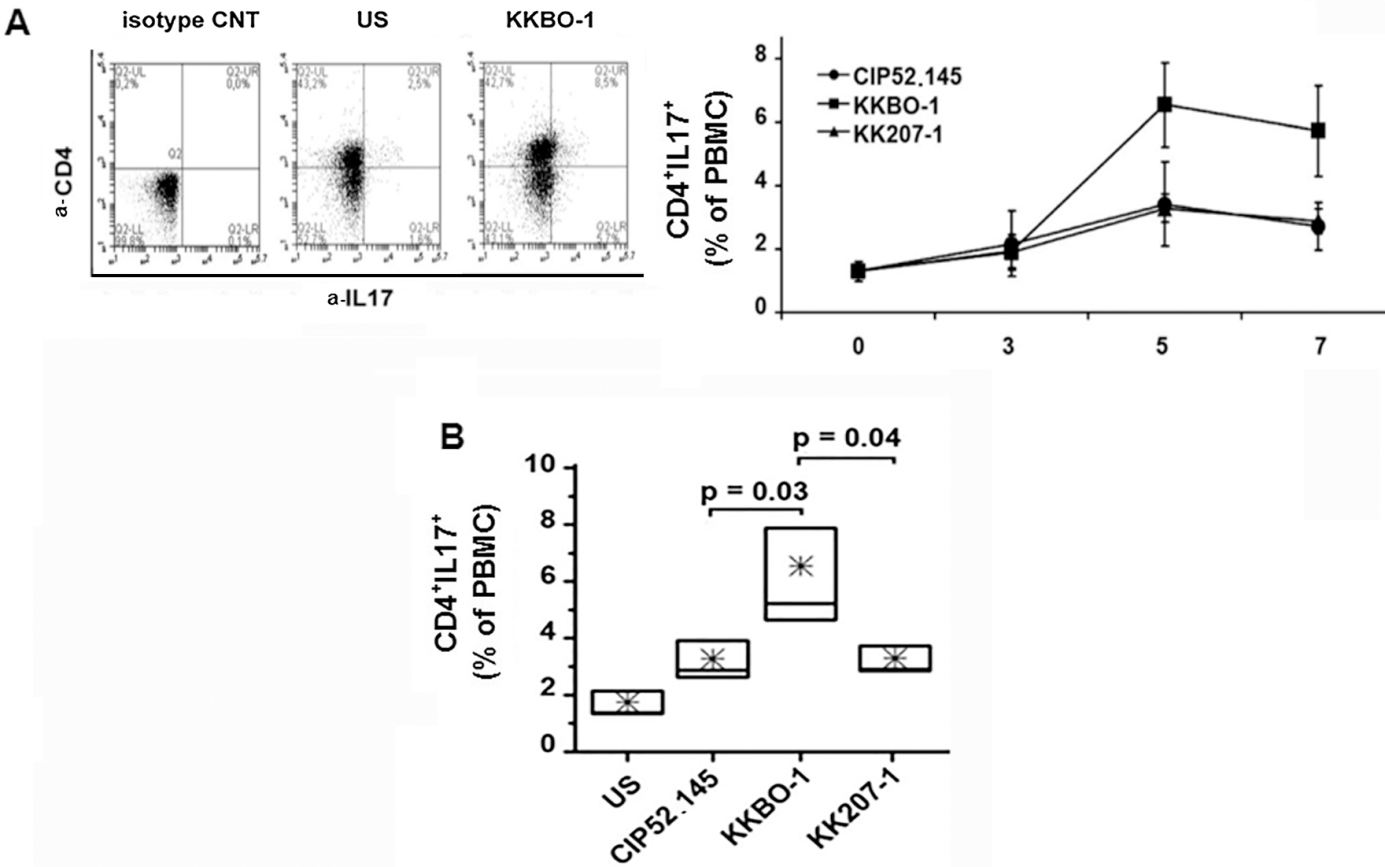
Differentiation of CD4^+^ T lymphocytes induced by *K*. *pneumoniae* strains. PBMC from 6 healthy donors were cultured at 10^6^/ml for 7 days with UV-inactivated bacterial cells at 1:10 ratio. Cells were recovered at the indicated times, stained with anti-CD4-APC and anti-IL-17A-FITC and analyzed by ACCURI instrument using the CflowPlus software to process the data. The area of positivity was determined by using an isotype-matched control mAb. Representative scatter plots at day 5 are shown. Panel A: Time course analysis of Th17 differentiation. Data are expressed as percentage of CD4^+^IL17^+^ (mean ± SE). Panel B: Percentage of TH-17 cells after 5 days of culture with bacterial cells (n = 6). The boxes extend from SE, with a horizontal line at the median. An asterisk indicates the mean value. Statistical analysis was performed by Student’s *t*-test and One-Way ANOVA and *p* ≤ 0.05 was considered significant.

The percentage of CD4^+^IFNγ^+^ and CD4^+^IL-17A^+^ was recorded at different times (3, 5 and 7 days) as a marker of Th1 and Th17 differentiation, respectively. Cell viability, assessed by trypan blue exclusion at each time point, did not reveal significant differences among bacteria-stimulated cultures (data not shown). In agreement with data reported in the literature, obtained with different *K*. *pneumoniae* strains [17,32], we found a significant differentiation of Th17 effectors in PBMC cultures with UV-inactivated bacterial cells after 3 days of culture. Such increase reached its maximum after 5–7 days of incubation (Fig 2 panel A). Fig 2 panel B shows that, based on the percentage of CD4^+^IL17^+^, KKBO-1 was the most efficient stimulus for the Th17 differentiation either compared to the KK207-1 ST258 clade 1 strain or to the highly virulent CIP 52.145 strain. Comparable results were obtained by using heat-inactivated bacterial cells as a stimulus for Th differentiation (S1 Fig).

**Fig 3:**
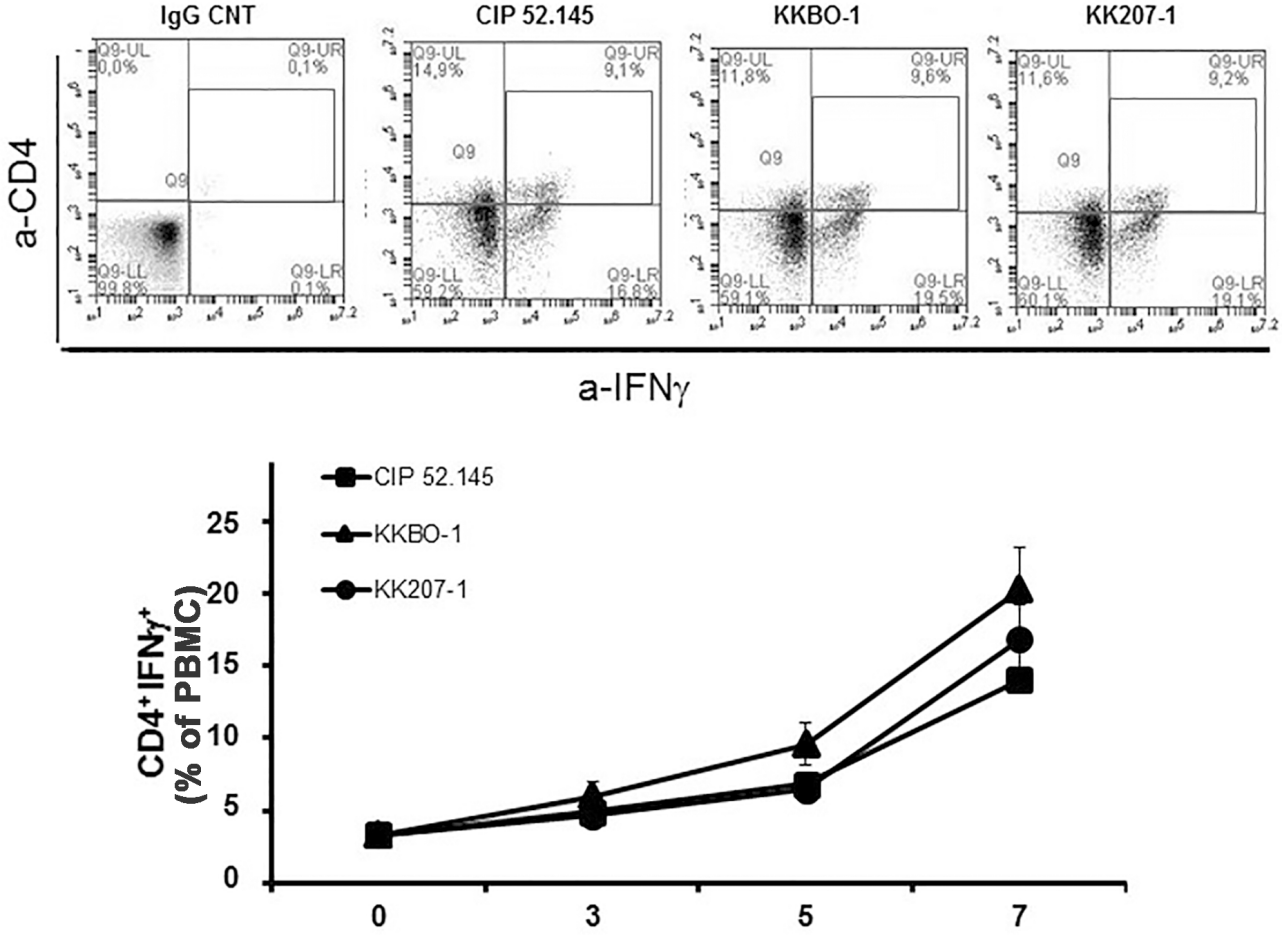
Th1 differentiation of CD4^+^ T lymphocytes induced by *K*. *pneumoniae* strains. PBMC from 6 healthy donors were cultured at 10^6^/ml for 7 days with UV-inactivated bacterial cells at 1:10 ratio. Cells were recovered at the indicated times, stained with anti-CD4-APC and anti-IFNγ-PerCP and analyzed by ACCURI instrument using the CflowPlus software to process the data. The area of positivity was determined by using an isotype-matched control mAb. Representative scatter plots at day 5 are shown. Data are expressed as percentage of CD4^+^IFNγ^+^ (mean ± SE).

As reported above, we also evaluated, in the same culture conditions, the amount of Th1 differentiation induced by UV-inactivated *K*. *pneumoniae* strains in PBMC from healthy donors. To this purpose, we evaluated the percentage of CD4^+^IFN-γ^+^ by cytofluorimetric analysis. Fig 3 shows the time course analysis of Th1 differentiation evaluated in PBMC cultures. Compared to time 0, the percentage of Th1 cells, evaluated as CD4^+^T cells producing IFN-γ, significantly increased after 5 days of incubation with bacterial cells and further increased after 7 days of culture. KKBO-1 appeared to be the highest inducer of differentiation in Th1 effectors. However, the observed differences were not statistically significant.

### Pathogen-induced Treg differentiation

**Fig 4:**
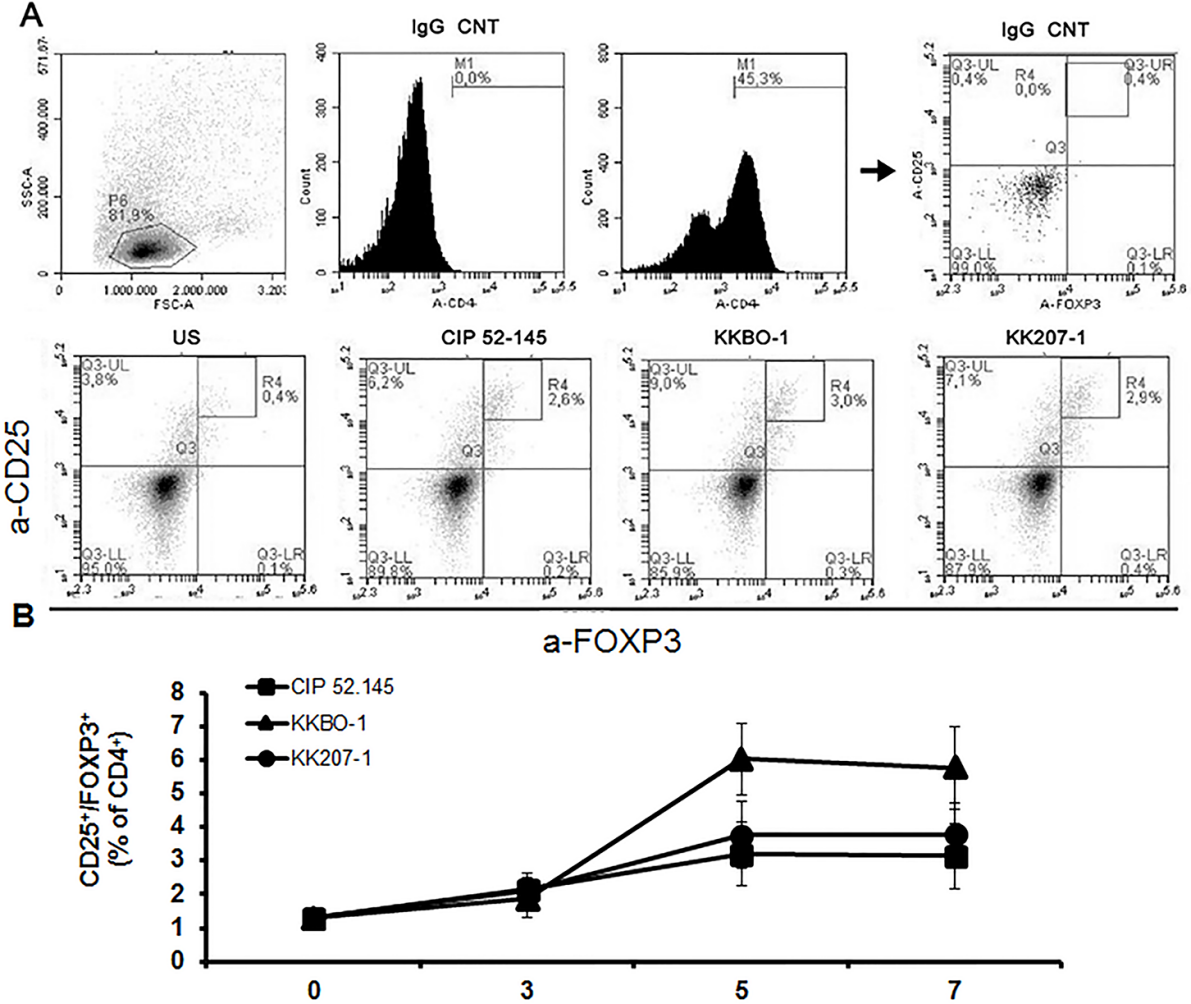
T regulatory cells induction in PBMC cultured with *K*. *pneumoniae* strains. Frozen PBMC from 6 healthy donors (already included in the study) were thawed and cultured at 10^6^/ml with UV-inactivated bacterial cells at 1:10 ratio. At time 0 and after 3, 5, 7 days of culture cells were stained with and anti-CD25-PE antibodies followed by intracellular staining with anti-Foxp3-APC antibodies and analyzed by ACCURI instrument, using the CflowPlus software to process the data. The area of positivity was determined by using an isotype-matched control mAb. Representative scatter plots at day 5 are shown in Panel A. Panel B show the time-course analysis of T regulatory cells (CD4^+^CD25^high^Foxp3^+^) differentiation in the PBMC population. Data are reported as mean percentage ± SE of 6 different experiments. Statistical analysis was performed by One-Way ANOVA and *p* ≤ 0.05 was considered significant; no significant differences were revealed.

Finally, to complete the immunological study, we investigated whether the two ST258 strains and CIP 52.145 differently induced the expansion of Treg cells. We cultured PBMC from 6/7 donors included in the study with bacterial cells and evaluated the expansion of Treg cells by measuring the percentage of CD4^+^CD25^high^Foxp3^+^ cells after 3, 5 and 7 days of culture. The percentage of CD4^+^IL17^+^ was also assessed in the same cultures. The results of these experiments (Fig 4) clearly showed that a Treg population is expanded following stimulation with bacterial cells from all the *K*. *pneumoniae* strains tested, with no significant differences among different strains at any time of culture. The kinetic of Treg differentiation was similar to that of Th17 response and we also confirmed Th17 differentiation in the same cultures (data not shown).

### Maturation of monocytes and myeloid dendritic cells induced by *K*. *pneumoniae* strains

To investigate the molecular mechanisms accounting for the observed differences in Th17 response induced by *K*. *pneumoniae* strains, the functional properties of monocytes and monocyte-derived dendritic cells (MDC) were studied before and after culture with the *K*. *pneumoniae* strains. First, we assessed whether monocytes and MDC were able to internalize bacterial cells from ST258 or CIP 52.145 *K*. *pneumoniae* strains. According to previous reports [15], the results of this study revealed that monocytes and MDC were able to internalize live bacterial cells of ST258 *K*. *pneumoniae* strains at comparable amounts (> 60%), and no significant differences were observed in internalization of ST258 or CIP 52.145 *K*. *pneumoniae* strains (S2 Fig).

**Fig 5:**
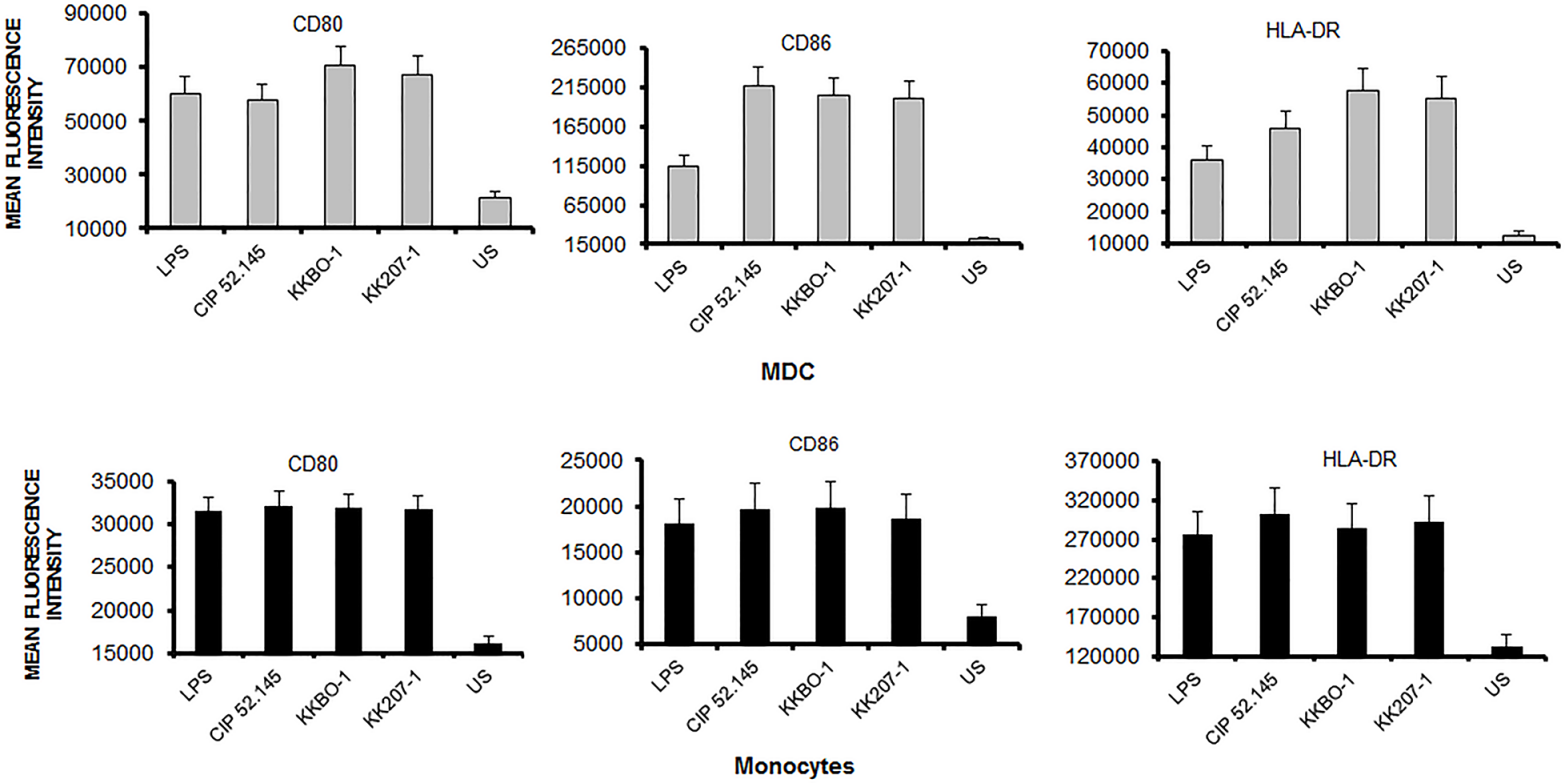
Effect of *K*. *pneumoniae* strains on the function of dendritic cells. MDC or monocytes were cultured for 16 hours with UV-treated bacteria or with LPS (400 ng/ml) as a control. At the end of incubation, MDC were stained with anti-CD80-FITC, anti-CD86-APC, anti-HLA-DR-PE or isotype control and analyzed by cytofluorimetric analysis. Results are expressed as mean fluorescence intensity. Data from 6 experiments (mean ± SE) are shown. Statistical analysis was performed by One-Way ANOVA and *p* ≤ 0.05 was considered significant; no significant differences were revealed.

Afterward, we studied the expression of determinants involved in antigen presentation. Monocytes and MDC were cultured with bacterial cells, either UV-inactivated or heat-inactivated, for 16 hours at 1:10 cell ratio. At the end of incubation, cells were stained with antibodies to CD80, CD86, HLA-DR and analyzed by cytofluorimetry. Fig 5 shows the results obtained using UV-inactivated bacteria: all strains induced ~ 2.5-fold increase in the expression of CD80, CD86 and HLA-DR compared to non-stimulated cultures. Comparable results were recorded by using heat-inactivated bacteria as a stimulant (data not shown).

**Fig 6:**
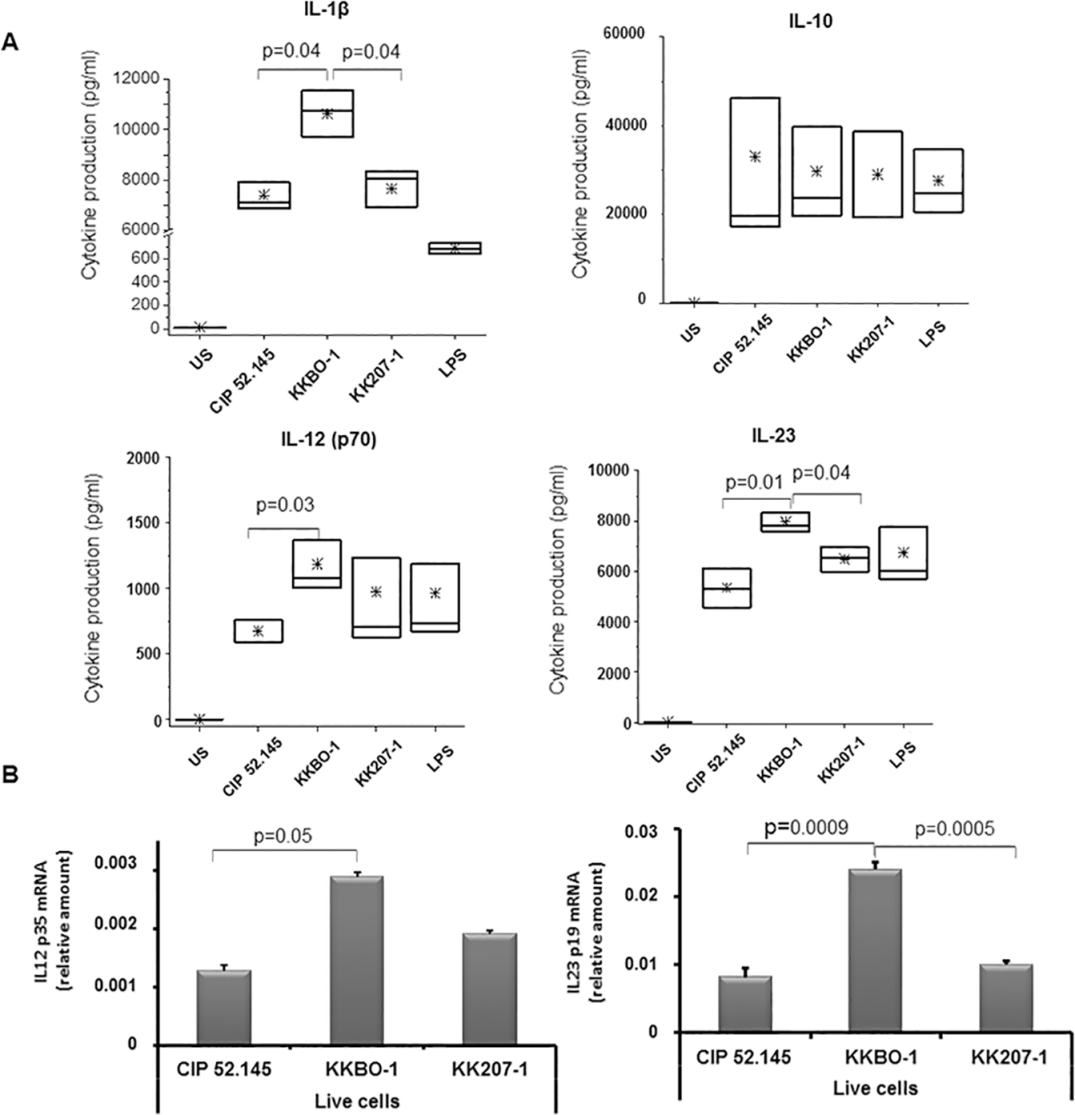
**Panel A. Cytokine production by MDC cultured with *K*. *pneumoniae* strains.** MDC from 6 different healthy donors were cultured with UV-inactivated bacterial cells at 1:10 cell ratio for 16 hours. Cytokine concentration was measured in culture supernatants by Immunoplex array. The boxes extend from SE, with a horizontal line at the median. An asterisk indicates the mean value. Statistical analysis was performed by Student’s *t*-test and One-way ANOVA. *p* ≤ 0.05 was considered significant. **Panel B. Expression of IL-12 and IL-23 mRNA by MDC cultured with live bacterial cells.** 10^6^ MDC (n = 3) were cultured with 10^6^live bacterial cells (MOI = 1) for 5 hours. At the end of culture, cells were lysed to obtain mRNA. The expression of IL-12 and IL-23 gene was evaluated by RT-PCR by using specific primers. Results are expressed as relative amounts (2^-ΔCT^) of mRNA. Data from 3 different experiments (mean ± SE) are shown. Statistical analysis was performed by Student’s *t*-test. *p* ≤ 0.05 was considered significant.

Finally, we evaluated the production of cytokines inducing T cell differentiation as IL-1β, IL-12, IL-23, IL-10 by MDC, a cell type mainly involved in the differentiation of Th1/Th17 cells and of Treg [33]. MDC were cultured with UV-inactivated bacterial cells for 48 hours and cytokine production was measured in culture supernatants. The results of these experiments, shown in Fig 6, confirmed the higher production of IL-1β induced by KKBO-1 compared to KK207-1 [15]. Fig 6, panel A shows that KKBO-1 also induced the production of significantly higher amounts of IL-23 compared to KK207-1 and CIP 52.145. The production of IL-12 induced by KKBO-1 was also higher, but statistical significance was reached only in comparison with CIP 52.145. The production of IL-10 was equally induced by all *K*. *pneumoniae* strains. Comparable results were obtained by using heat-inactivated bacterial cells at 1:10 cell ratio as a stimulant for cytokine production (data not shown).

Finally, to confirm the results about the MDC production of cytokines involved in Th1/Th17 differentiation, we performed cultures of MDC with live bacterial cells at an MOI = 10 for shorter times (4–8 hours) and monitored the production of mRNA for IL-23 and IL-12. Fig 6 panel B shows that *IL-23* gene was significantly more expressed in MDC cultured with KKBO-1 compared to KK207-1 or CIP 52.145. *IL-12* gene was more expressed in cultures with KKBO-1 but the differences reached statistical significance only in comparison with cultures stimulated with CIP 52.145.

The clonal expansion of members of the CG258 clonal lineage has largely contributed to the global diffusion of KPC-producing *K*. *pneumoniae*, with strains of clade 2 being apparently more prevalent [10,12,13]. Relatively little is known about the reasons for this epidemiological success, but their fitness in the human reservoir suggests some evolutionary advantage. Competition with microbial commensals and peculiar interactions with host immunity are likely to be involved in the successful spread of CG258 *K*. *pneumoniae* and of clade 2 strains in particular [34].

Recent data have shown a very limited binding and uptake of CG258 *K*. *pneumoniae* strains by human neutrophils, not dependent on the clade-specific CPS type [35], and evidence has been reported on the ability of selected strains to evade and inhibit host innate immune clearance from lung tissue [36]. We have previously studied the ability of strains representative of clade 2 and clade 1 to activate the production of inflammatory cytokines by cells of innate immunity and revealed differences in the ability to induce the production of IL-1β dependent on CPS type: in particular strains expressing CPS-2 (clade 2) more efficiently induce the production of IL-1β by monocytes and dendritic cells through the NLRP3 inflammasome pathway [15].

These results prompted us to investigate whether strains of the two clades of CG258 differently induce adaptive immune responses. Indeed, it is known that the role of IL-1β in the immune response against a pathogen is not limited to the induction of the inflammatory process [37]. IL-1β enhances the expansion of CD4 T cells and promote their differentiation particularly into IL-17-producing effector T cells [38]. TH17 cells produce cytokines that induce the neutrophil recruitment and the release of lipocalin-2 and of anti-microbial peptides by mucosal epithelial cells, providing efficient defenses against extracellular pathogens [39]. Furthermore, they potentiate Th17-driven Th1 response [39] providing immunity against a broad range of pathogens including the hypervirulent strains of *K*. *pneumoniae* [18].

Using an *ex-vivo* antigen-pulse of PBMC associated with the intracellular cytokine staining of T lymphocyte we revealed an increase in the percentage of Th17 (CD4^+^IL17^+^) and of Th1 (CD4^+^IFN-γ^+^) cells in most of bacterial-stimulated PBMC cultures. While the expansion of Th1 populations was not differently induced by the *K*. *pneumoniae* strains, significant differences emerged in the induction of TH17 differentiation. KKBO-1 (ST258 clade 2) was the most potent inducer of Th17 differentiation compared to either the KK207-1 (ST258 clade 1) or the highly virulent CIP 52.145 strain. Accordingly, cytokine measurement in supernatants of bacteria-stimulated cultures revealed that KKBO-1 was the most potent inducer of TNF-α and IL-17A. The kinetic of TH-17 response was more similar to a memory recall response rather than priming and activation of naïve T lymphocytes. This was not surprising since the adaptive immune response and selectively the activation of mucosal Th17 lymphocytes is usually directed against antigenic determinants, (derived from surface proteins as OMP, fimbriae etc), common to different clones of *K*. *pneumoniae* diffused in the environment and often colonizing the intestinal tract of healthy individuals [16]. The molecular mechanisms at the basis of the differential TH17 response likely reside in the functional properties of antigen-presenting cells induced by bacterial components, including but not limited to proteic antigens.

In this context, the capsular composition, which represents the major divergence between the clades, [10,11] may have a role in the adaptive immune response through the modulation of the molecular pathways leading to IL-1β production [15] in antigen-presenting cells. Indeed, it is known that the production of IL-23 by antigen-presenting cells and the expression of IL-23 receptor on T cells are induced by IL-1β [22,23]. According to with this hypothesis, we found significant differences in IL-23 gene expression and in the production of IL-23 by MDC cultured with KKBO-1 or KK207-1.

Altogether, these results suggest that CG258-KP of the two clades induce a different adaptive immune response in humans, with strains of clade 2 apparently endowed with the highest immuno-inflammatory properties.

Our data suggest that the selective pressure of adaptive immune response may have allowed the survival of phenotypes with lower immunogenic property and perhaps higher virulence (clade 1). On the other side, triggering of inflammation with the production of selected cytokines as TNF, which disrupt epithelial tight junctions and impair gut barrier integrity [40,41], may be used by strains of clade 2 to disseminate to deeper tissues. These events may occur in gut-colonized patients after treatments inducing marked intestinal dysbiosis and might be responsible for the predominance of this clade among CG258 *K*. *pneumoniae* strains isolated from invasive infection (13). Further experiments, including an epidemiological analysis of the prevalence of cps-1 and -2 in CG258-KP colonized patients will be needed to confirm this hypothesis.

## Supporting information

S1 Fig Cytokine production by PBMC stimulated with heat-inactivated bacterial cells. Panel A. PBMC were isolated from 6 healthy donors and cultured at 10^6^/ml for 7 days with heat-inactivated bacterial cells from the ST258 KP-KPC strains or from CIP 52.145. LPS was used as an internal standard at 400 ng/ml. Cytokine concentration was measured in culture supernatants collected after 5 days of culture by Immunoplex array. The boxes extend from SE, with a horizontal line at the median. An asterisk indicates the mean value. Statistical analysis was performed by Student’s t-test and One-Way ANOVA and p ≤ 0.05 was considered significant. Panel B. PBMC were isolated and cultured as described above. Cells were collected at 3, 5 and 7 days, stained with anti-CD4-APC followed by intracellular staining with anti-IL-17A-FITC and analyzed by ACCURI instrument. Data were processed through CflowPlus software. Ten thousand events for each sample were acquired. The area of positivity was determined by using an isotype-matched control mAb. The box-chart plots shows the percentage of Th-17 cells at day 5. The boxes extend from SE, with a horizontal line at the median. An asterisk indicates the mean value. Results from 6 different experiments are shown. Statistical analysis was performed by Student’s t-test and One-Way ANOVA and p ≤ 0.05 was considered significant.
(TIF)

S2 Fig Internalization of K. pneumoniae strains by human monocytes. *K*. *pneumoniae* cells were incubated for 45 minutes at 37°C with human monocytes at 10:1 cell ratio. At the end of the incubation, monocytes were extensively washed, lysed by 0.5% tritonX-100 and plated on Mueller-Hinton Agar to measure bacterial survival. CFU counts were performed after 24 hours. Results are shown as percentage of internalized bacteria (mean ± SE) over total bacteria added to the culture. Data from 3 different experiments (mean ± SE) are shown.
(TIF)

S1 Table Cytokine production by PBMC cultured with K. pneumoniae strains. PBMC were cultured at 10^6^cells/ml in the presence or absence of 10^7^ UV-inactivated bacterial cells for 5 days. Cytokines were measured in culture supernatants through Immunoplex array.
(DOCX)
