# Supplementary figures and images for: Differential Th17 response induced by the two clades of the pandemic ST258 Klebsiella pneumoniae clonal lineages producing KPC-type carbapenemase
Source: PLoS One. 2017 Jun 6;12(6):e0178847. doi: 10.1371/journal.pone.0178847 (PMC5460819; doi:10.1371/journal.pone.0178847)

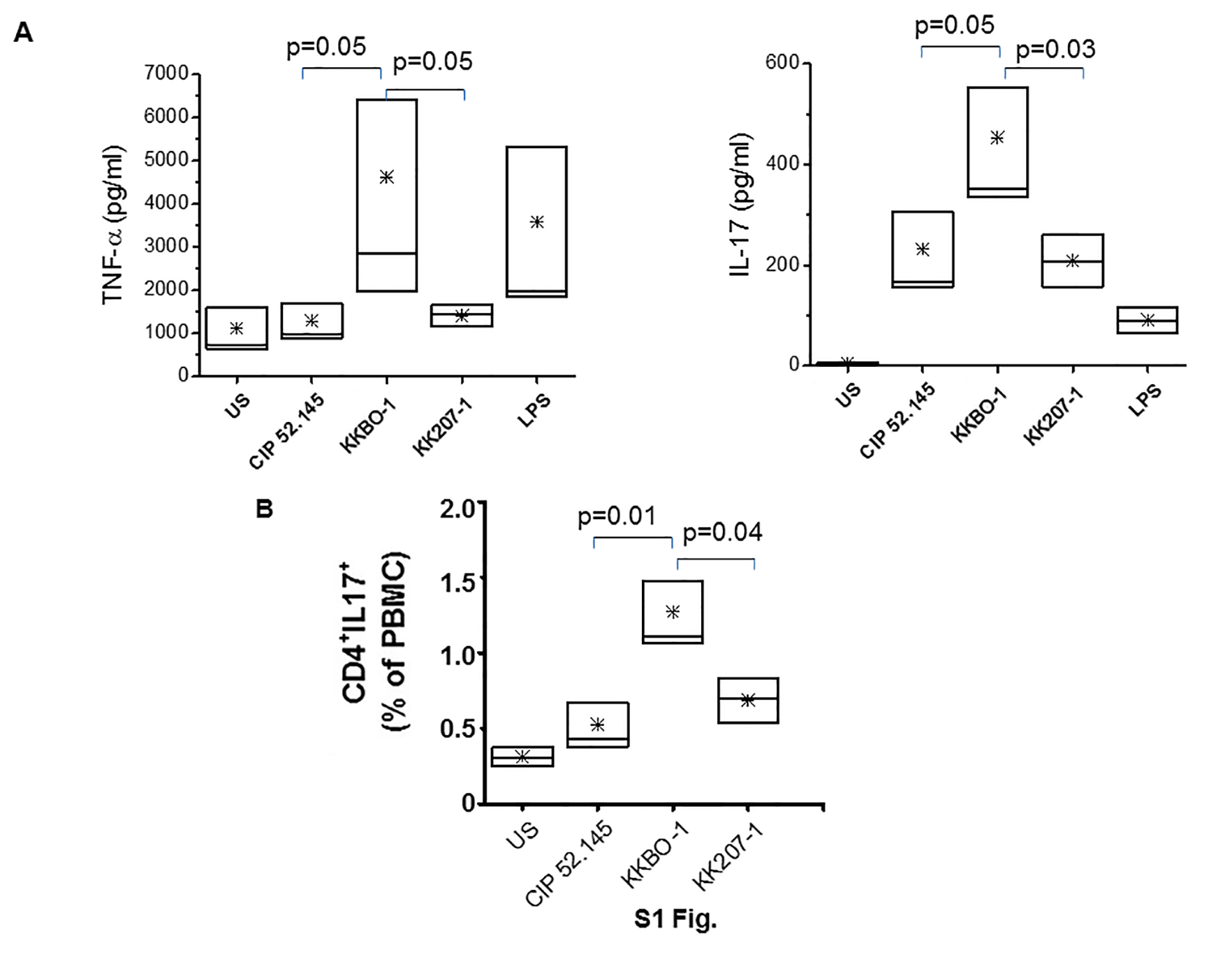

Supplement: S1 Fig — Panel A. PBMC were isolated from 6 healthy donors and cultured at 106/ml for 7 days with heat-inactivated bacterial cells from the ST258 KP-KPC strains or from CIP 52.145. LPS was used as an internal standard at 400 ng/ml. Cytokine concentration was measured in culture supernatants collected after 5 days of culture by Immunoplex array. The boxes extend from SE, with a horizontal line at the median. An asterisk indicates the mean value. Statistical analysis was performed by Student’s t-test and One-Way ANOVA and p ≤ 0.05 was considered significant. Panel B. PBMC were isolated and cultured as described above. Cells were collected at 3, 5 and 7 days, stained with anti-CD4-APC followed by intracellular staining with anti-IL-17A-FITC and analyzed by ACCURI instrument. Data were processed through CflowPlus software. Ten thousand events for each sample were acquired. The area of positivity was determined by using an isotype-matched control mAb. The box-chart plots shows the percentage of Th-17 cells at day 5. The boxes extend from SE, with a horizontal line at the median. An asterisk indicates the mean value. Results from 6 different experiments are shown. Statistical analysis was performed by Student’s t-test and One-Way ANOVA and p ≤ 0.05 was considered significant. (TIF) [file pone.0178847.s001.tif]

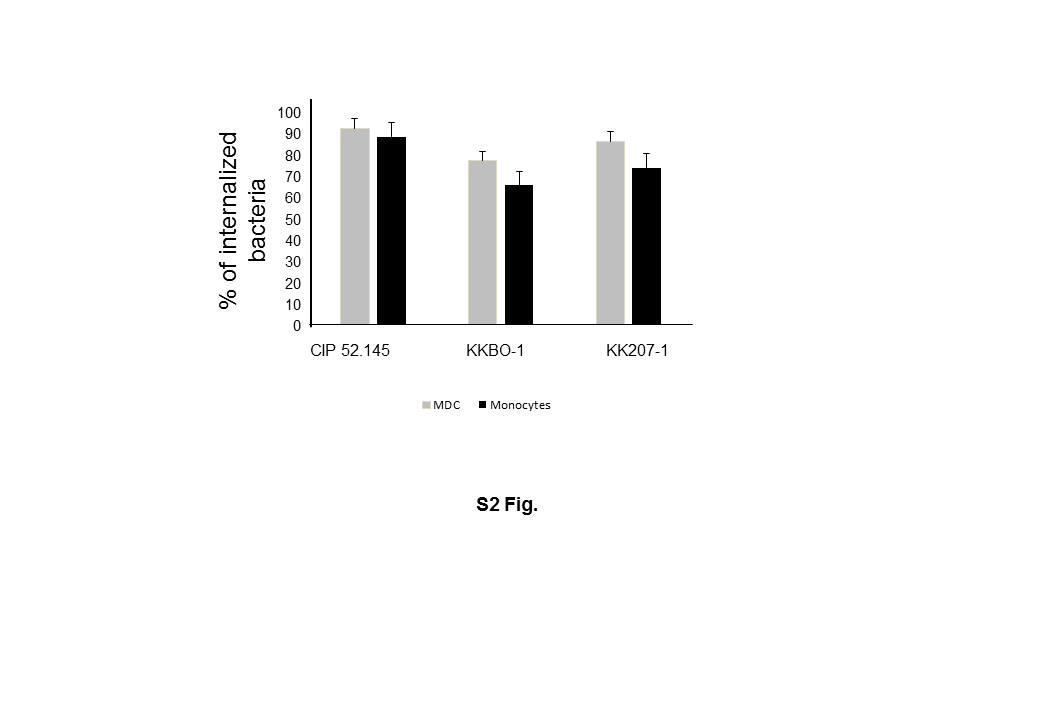

Supplement: S2 Fig — K. pneumoniae cells were incubated for 45 minutes at 37°C with human monocytes at 10:1 cell ratio. At the end of the incubation, monocytes were extensively washed, lysed by 0.5% tritonX-100 and plated on Mueller-Hinton Agar to measure bacterial survival. CFU counts were performed after 24 hours. Results are shown as percentage of internalized bacteria (mean ± SE) over total bacteria added to the culture. Data from 3 different experiments (mean ± SE) are shown. (TIF) [file pone.0178847.s002.TIF]
