# Supplementary material for: Differential Th17 response induced by the two clades of the pandemic ST258 Klebsiella pneumoniae clonal lineages producing KPC-type carbapenemase
Source: PLoS One. 2017 Jun 6;12(6):e0178847. doi: 10.1371/journal.pone.0178847 (PMC5460819; doi:10.1371/journal.pone.0178847)
Supplement: S1 Table — PBMC were cultured at 106cells/ml in the presence or absence of 107 UV-inactivated bacterial cells for 5 days. Cytokines were measured in culture supernatants through Immunoplex array. (DOCX) [file pone.0178847.s003.docx]

| **Cytokine (pg/ml**) | **KKBO-1** | **KK207-1** | **CIP52.145** | **LPS** | **US** |
| --- | --- | --- | --- | --- | --- |
| **IL-1β** | 1949 ± 480 | 1158 ± 425 | 819± 195 | 659± 381 | 3± 1 |
| **IFN γ** | 6420± 3077 | 2561± 1258 | 1866± 927 | 1015± 506 | 34± 15 |
| **IL-10** | 10973± 1492 | 8917± 1993 | 10112± 1081 | 3941± 1423 | 60 ± 14 |
| **IL-6** | 5660 ± 1633 | 6245 ± 1077 | 7477± 1161 | 1852± 1459 | 2119± 761 |

**S1 Table**
